# Phototest for neurocognitive screening in multiple sclerosis

**DOI:** 10.1590/S1980-57642016DN10100003

**Published:** 2016

**Authors:** Joana Pinto, Emanuela Lopes, Gerly Gonçalves, Ângela Silva, Carnero-Pardo, Bruno Peixoto

**Affiliations:** 1 CESPU, Instituto de Investigação e Formação Avançada em Ciências e Tecnologias da Saúde, Gandra, Portugal; 2 Centro Hospitalar do Alto Ave, Guimarães, Portugal; 3 Cognitive Behavioral Neurology Unit of the Hospital Universitario Virgen de las Nieves, Granada, Spain; 4 FIDYAN Neurocenter, Granada, Spain; 5 CESPU, Instituto Superior de Ciências da Saúde - Norte (Sciences Department), Gandra, Portugal

**Keywords:** multiple sclerosis, Phototest, Montreal Cognitive Assessment, Expanded Disability Status Scale, Fatigue Severity Scale, esclerose múltipla, Fototest, Montreal Cognitive Assessment, Expanded Disability Status Scale, Escala de Severidade de Fadiga

## Abstract

Multiple Sclerosis (MS) is one of the most common neurological disorders. Cognitive dysfunction is considered a clinical marker of MS, where approximately half of patients with MS have cognitive impairment.

**Objective:**

The Phototest (PT) is a brief cognitive test with high diagnostic sensitivity, accuracy and cost-effectiveness for detecting cognitive deterioration. Our aim was to test the utility of the PT as a neurocognitive screening instrument for MS.

**Methods:**

The study enrolled 30 patients with different types of MS from an outpatient clinic as well as 19 healthy participants. In conjunction with the PT, the Montreal Cognitive Assessment (MoCA), Barthel Index (BI), Expanded Disability Status Scale (EDSS), and Fatigue Severity Scale (FSS) were administered.

**Results:**

The MS group obtained significantly lower results on all domains of the PT, except for the naming task. The PT showed good concurrent validity with the MoCA. In direct comparison to the MoCA, PT showed a greater area under the curve and higher levels of sensitivity and specificity for MS neurocognitive impairments. A cut-off score of 31 on the Phototest was associated with sensitivity of 100% and specificity of 76.7%.

**Conclusion:**

The PT is a valid, specific, sensitive and brief test that is not dependent on motor functions. The instrument could be an option for neurocognitive screening in MS, especially in identifying cases for further neuropsychological assessment and intervention.

## RESUMO

A Esclerose Múltipla (EM) é das doenças neurológicas mais comuns. A disfunção cognitiva consiste num marcador clínico da EM, cerca de metade dos pacientes apresentam comprometimento cognitivo.

**Objetivo:**

O Fototest (FT) é um teste breve, sensível, específico e com boa relação custo-eficácia na deteção de deterioração cognitiva. Pretendemos testar a validade do FT como um instrumento de screening neurocognitivo na EM.

**Métodos:**

O estudo envolveu uma amostra de 30 doentes com diferentes tipos de EM de uma clínica de tratamento ambulatório e 19 participantes saudáveis. Em conjunto com o FT, foram aplicados o *Montreal Cognitive Assessment* (MoCA), o Índice de Barthel (IB), a *Expanded Disability Status Scale* (EDSS) e a Escala de Severidade de Fadiga (FSS).

**Resultados:**

O grupo EM obteve resultados significativamente inferiores em todos os domínios do FT, excepto na tarefa de nomeação. O FT apresenta boa validade concorrente com o MoCa. Na comparação direta com o MoCa, o FT revelou uma área sob a curva superior e níveis de sensibilidade e especificidade para os défices cognitivos na EM superiores. Ao ponto de corte de 31 no FT correspondem valores de sensibilidade de 100% e especificidade de 76,7%.

**Conclusão:**

O FT é um teste válido, específico, sensível e breve, não dependente das funções motoras. Pode ser uma opção para o *screening* neurocognitivo na EM, especialmente na identificação de casos para posterior avaliação neuropsicológica e intervenção.

## INTRODUCTION

Cognitive dysfunction is a clinical marker of MS^1^ and encompasses all stages and types of clinical progression. These cognitive deficits lead to limitations in work and social life, independently of the degree of physical disability.^2^ Cognitive impairment affects up to 65% of patients and can occur from the early stages of the disease, tending to worsen over time.^2^^,^ 3

General intellectual functioning is preserved in the majority of patients,^4^ despite significant impairment in fluid intelligence.^5^ The processing speed of visual and auditory information and verbal fluency are the cognitive domains affected earliest.^1^^,^ 3 The decrease in processing speed represents the most prominent and common cognitive sign in MS and is intimately associated with the severity of the disease.^6^ The decrease in processing speed also impairs working memory encoding.^7^ Deficits in semantic and phonologic verbal fluency are also frequent among MS patients.^8^ Verbal fluency seems to be impaired at early stages of relapsing/remitting MS, and this impairment increases with MS duration.^9^ In fact, verbal fluency and processing speed tasks may be amongst the most sensitive neuropsychological measures to cognitive impairment in MS.^8^

With progression of the disease, memory deficits, particularly in recall and delayed recall,^3^ become obvious. Furthermore, MS patients show deficits in working memory,^1^^,^ 7 long-term memory, nonverbal memory, visuospatial memory^10^ and in autobiographic memory.^5^

Executive functioning is impaired in the ability to solve problems,^5^^,^ 10 in abstract reasoning,^10^ planning, organization, rule change, inhibition and verbal fluency.^5^ Deficits in divided attention, sustained attention and in focal attention^5^ are also frequent.

Visuoconstructive and visuoperceptive abilities are also affected, particularly in color discrimination and in the perception of the Müller-Lyer illusion, as well as in visuospatial integration and discrimination and on complex tasks of facial recognition.^5^

Language deficits are not common,^5^ although some authors have reported naming difficulties.^11^

Cognitive assessment of MS patients is the first step for the early detection of neurocognitive impairment and for the implementation of therapeutic measures to prevent further decline and decrease the impact of deficits on patients' daily life. However, this assessment is not performed routinely due to the lack of tests that are sensitive , simple, easy-to-administer and interpret, and cost-effectivene.^12^

The Phototest (www.fototest.es) is a brief (<3 minutes) cognitive test that is easy to administer and assesses several cognitive domains (language, episodic memory and verbal fluency). It has shown high diagnostic sensitivity, accuracy and cost-effectiveness for detecting cognitive deterioration in the context of mild cognitive impairment. Considering costs based on public prices and hospital accounts, the costs involved with the use of the Phototest are considerably lower in comparison with other screening tests. Because reading is not required and there are no pencil and paper tasks, this test is suitable for use with illiterate subjects or individuals with a low level of education.^13^

Given the clinical characteristics of MS, we aimed to test the suitability of the Phototest as a neurocognitive screening instrument in the context of MS. Therefore, the discriminant validity, sensitivity and specificity of the Phototest were determined, as well as its concurrent validity and relationship to clinical variables. 

## METHODS

**Participants.** The sample comprised two groups: a clinical group of 30 subjects (19 women and 11 men) with an MS diagnosis, and a control group of 19 healthy subjects (14 women and 5 men). The MS group had a mean age of 40.47 ± 11.1 years and the control group had a mean age of 37.68 ± 12.09 years. Mean years of education in the MS group was 10.8 ± 5.5 and in the control group was 11.42 ± 5.35. Patients were recruited at the neurology outpatient clinic of the *Centro Hospitalar do Alto Ave,* and the subjects in the control group were blood donors. The study had the approval of the *Centro Hospitalar do Alto Ave* Ethics Committee.

Individuals with prior history of neuropsychiatric or systemic pathologies liable to directly interfere in neurocognitive functioning were excluded. Alcohol or drugs abuse, illiteracy and uncorrected sensory-perceptive impairments also constituted exclusion criteria. Illiterate participants were excluded because illiteracy would interfere with the performance on the MoCA

To assure that the control group was cognitively intact, individuals scoring ≤ 1 standard deviation on the Montreal Cognitive Assessment were excluded from the sample, in accordance with the Portuguese norms for the test regarding age and education.

Groups did not differ for age (t = -2.013; p = .485), gender (c2 = .567; p = .541) or education (t = 1.106; p = .504).

### Study measures

Phototest. The Phototest is a brief , easy-to-administer cognitive test that comprises three parts: a naming task with six color photographs of common objects; a categorical verbal fluency task in which subjects must evoke male and female names; and free and cued recall of the six objects used in the naming task. This test was developed in Spain and has shown high diagnostic accuracy and effectiveness in the context of cognitive impairment and dementia, even compared to more traditional screening tests such as the Mini-Mental State Examination and the Memory Alteration Test.^13^ It has been demonstrated that cut-off points of 26 and 28 provide satisfactory discriminant validity for dementia and cognitive impairment, respectively The Phototest also has good test-retest and inter-observer reliability. The test has normative data and some psychometric characteristics for the Portuguese population.^14^

Montreal Cognitive Assessment. The Montreal Cognitive Assessment (MoCa) it is a cognitive screening test that assesses several cognitive domains, including: executive functions through an abbreviated form of the trail-making test part B (TMT B); visuospatial abilities through the copy of a 3-dimensional cube (Cube) and the clock drawing task (Clock); language is assessed by the naming task of three animals (Naming), the repeating of two complex phrases (Phrases) and a phonetic verbal fluency task (Verbal Fluency); attention and concentration are assessed using direct and indirect digit span (Digits), cancelation (Canceling) and serial subtraction (Subtraction) tasks; abstract thinking is assessed by a similarities task (Similarities); memory through the learning and recall of 5 words (delayed recall); temporal and spatial orientation are also assessed using six questions. This test was used because it has high sensitivity to neurocognitive impairments in MS^15^ making it a good instrument for establishing the concurrent validity of Phototest. MoCA was also used to guarantee the cognitive normality of the subjects in the control group.

Fatigue Severity Scale. The Fatigue Severity Scale (FSS) is a self-report scale that assesses the perception of fatigue of MS patients in physical functioning, exercise, work, family and social life. It has good psychometric properties and high construct validity.^16^ The FSS was used in order to characterize the clinical sample and correlate it with Phototest.

Barthel Index. The Barthel Index (BI) evaluates 10 activities: feeding, grooming, toilet use, bathing, dressing, sphincter control, walking, transfers, and stair climbing. This test has shown good psychometric qualities for evaluating functionality in activities of daily living in Portuguese patients.^17^

Expanded Disability Status Scale (EDSS). Given the positive correlation between neurological disability and cognitive functioning, the Expanded Disability Status Scale (EDSS) was used to test Phototest sensitivity to neurological disability. EDSS is the most well-known and widely used scale for quantifying the degree of disability in MS.^18^ EDSS assesses eight functional systems: pyramidal, cerebellar, brainstem, sensorial system, bowel and bladder, visual and cerebral.^19^ The results can range from 0 (normal) to 10 (death due to MS).^19^ EDSS has good inter and intra-observer reliability and face validity with other disability scales.^18^ Results on the EDSS were used to determine the degree of neurological disability of the clinical group and to correlate it with the results on the Phototest.

**Procedure.** This study was approved by the Research Ethics Committee of the *Centro Hospitalar do Alto Ave* and all participants gave their informed consent. All subjects were assessed using the Phototest and the MoCA. The IB, FSS and EDSS were applied only to the clinical group. The neuropsychological assessment was conducted in a closed room and took approximately fourteen minutes.

### **Statistical analysis.** Statistical analysis was carried out using the program IBM Statistics version 21 for Windows.

Central tendency and deviation measures were used to analyze the sample characteristics and results obtained. The comparison of test performance between groups was performed using the Mann-Whitney U test. The sensitivity and specificity of the Phototest were determined by a Receiver Operating Curve (ROC). The concurrent validity between the Phototest and the MoCA was calculated by the Spearman correlation coefficient.

A value of p<.05 was considered statistically significant.

## RESULTS

**Clinical characteristics.** Table 1 shows the clinical characteristics of the MS group. The majority of the sample comprised patients with relapsing-remitting MS. All patients were in current use of medication for MS. In general, the sample revealed a moderate level of disability and was functional for activities of daily living, despite reporting moderate levels of fatigue.

**Table 1.** Clinical characteristics of the MS group.

		**MS (N = 30)**
Patterns of progression (n / %)	SPMS	5 / 10.2%
	RRMS	24 / 49.0%
	PPMS	1 / 2.0%
Medication (n / %)	Pain medication	7 / 23%
	Anxiolytics	6 / 20%
	Antidepressants	6 / 20%
	Others	5 / 16.7%
Numbers of relapses (M (SD))		8.24 (11.28) [0-60]	
Years of MS (M (SD))		11.17 (8.2) [1.5-36]	
BI		93.67 (11.96)	
FSS		44.4 (11.77)	
EDSS		3.97 (2.57)	

SPMS: Secondary Progressive Multiple Sclerosis; RRMS: Relapsing-remitting Multiple Sclerosis; PPMS: Primary Progressive Multiple Sclerosis; BI: Barthel Index; FSS: Fatigue Severity Scale; EDSS: Expanded Disability Status Scale.

**Comparison between MS patients and controls.** MS patients revealed significantly lower performance on both neurocognitive tests and on the majority of the tasks. The naming tasks of both Phototest and MoCA tests, as well as the digit span, cancelling, phrases repeating, similarities and orientation tasks of MoCA, exhibited no significant differences between groups (Table 2). 

**Table 2.** Comparison of results obtained by the two groups on neuropsychological tests.

		**Control Group (n=19) M (SD)**	**MS Group (n=30) M (SD)**	**U**	**p**
Phototest	Naming	5.26 (0.45)	5.37 (0.56)	317	.434
	Free Recall	11.05 (01.39)	9 (2.02)	118.5	≤.001
	Cued Recall	0.47 (0.69)	1.30 (0.92)	429	.002
	Fluency Men	14.79 (3.31)	10.63 (3.23)	109	≤.001
	Fluency Women	14.79 (3.17)	11.27 (3.39)	126.5	.001
	Total	46.47 (5.74)	37.57 (7.01)	99	≤.001
MoCA	TMT B	0.95 (0.23)	0.53 (0.51)	167	.002
	Cube	0.84 (0.38)	0.37 (0.49)	149.5	.001
	Clock	2.89 (0.46)	2.03 (0.77)	107	≤.001
	Naming	2.84 (0.38)	2.6 (0.62)	232	.158
	Digits	1.84 (0.38)	1.6 (0.56)	224	.111
	Canceling	0.89 (0.32)	0.90 (0.31)	286.5	.953
	Subtraction	2.89 (0.32)	2.27 (0.91)	175	.006
	Phrases	1.53 (0.61)	1.6 (0.62)	307	.594
	Verbal Fluency	0.63 (.49)	0.27 (0.45)	181	.012
	Similarities	1.42 (0.69)	1.2 (0.71)	236	.273
	Delayed recall	3.05 (1.13)	2.17 (1.56)	196	.061
	Orientation	5.95 (0.23)	5.77 (0.63)	252	.234
	Total	25.74 (1.69)	21.27 (4.28)	107	≤.001

**Results of analysis of sensitivity and specificity of Phototest.** The results on Phototest showed a moderate positive correlation with MoCA results (r = 0.589; p = .000). None of the neurocognitive tests showed any correlation with either the FSS or EDSS. The same was observed regarding the number of relapses and duration of disease.

**Figure 1.**
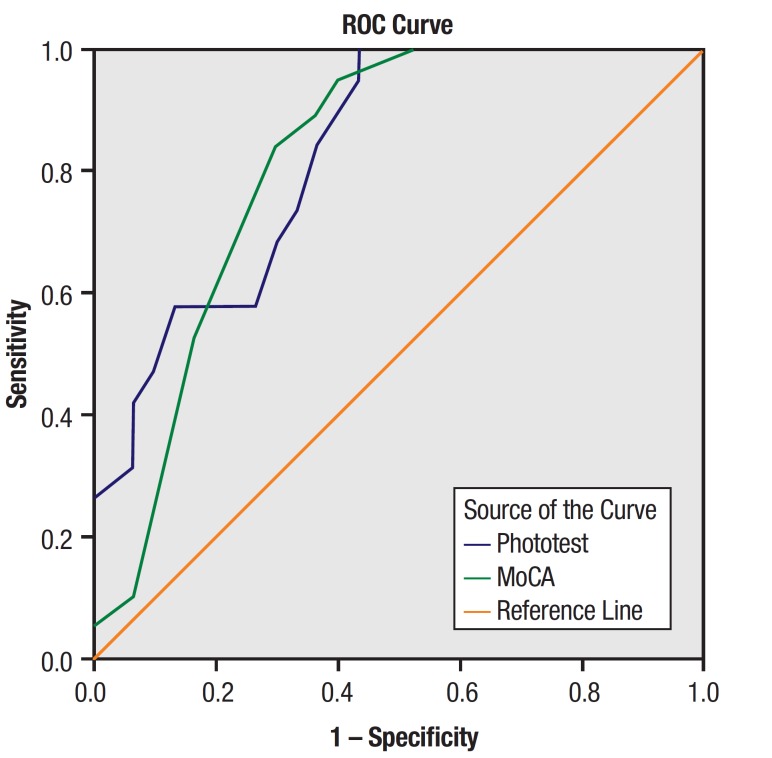
Receiver operating curve generated by the two neurocognitive tests.

The Phototest had an area under the curve of .826 (S.E. = .57; p = .000), slightly higher than the area under the curve generated for the MoCA (AUC = .81; S.E. = .061; p = .000), in distinguishing patients from controls (Figure 1).

Based on a cut-off of 31 points, the Phototest had sensitivity of 100% and specificity of 76.7%. A cut-off of 24 points on the MoCA represented sensitivity of 89.5% and specificity of 36.7%. These cut-off points represent two standard deviations according to the Portuguese normalization studies of these two tests, based on the mean age and years of schooling of the present sample. Several cut-off points and sensitivity and specificity values for both tests are displayed in Table 3.

**Table 3.** Cut-off points and corresponding sensitivity and specificity for Phototest and MoCA.

**Test**	**Cut-off points**	**Sensitivity**	**Specificity**
Phototest	25	100	0
	27	100	23.3
	29	100	36.7
	31	100	66.7
	32	100	76.7
	33	100	77.3
	34	100	79.4
	35	100	80.1
	36	100	82
	37	100	83.1
	38	100	84.4
	39	94.7	86.2
	40	89.5	88
	41	84.2	90.4
	42	73.7	96.2
	43	68.4	100
MoCA	15	100	0
	17	100	0
	18	100	6.7
	19	100	13.3
	20	100	16.7
	21	100	30
	23	94.7	34.5
	24	89.5	36.7
	25	84.2	53.3
	26	52.6	60
	27	36.8	73.3
	28	10.5	80
	29	5.3	83.3
	30	0	100

## DISCUSSION

The Phototest proved to be a sensitive and specific instrument for assessing general neurocognitive functioning in MS. Although the naming task failed to distinguish the two groups, the total score of the test revealed higher values of sensitivity and specificity compared to the MoCA. However, performance on the Phototest showed no association with fatigue, disability in activities of daily life living, disability or duration of the disease.

Our results showed good concurrent validity with the MoCA, a more extensive test for neurocognitive screening in MS.^15^ Perhaps the existence of two tasks of verbal fluency in the Phototest contributed to this finding. As previously stated, verbal fluency is one of the neurocognitive functions affected earliest in MS.^8^^,^ 9 Furthermore, the transition between the two tasks of verbal fluency in the Phototest requires verbal inhibitory control, as well as rule change. Thus, verbal fluency tasks in the Phototest indirectly encompass executive components implicated in MS.^5^ The implicit executive components in verbal fluency are highly influenced by processing speed,^20^ another cognitive domain commonly affected in MS.^6^

Processing speed and executive functioning are the main predictors of performance in episodic memory.^21^ This observation may account for the finding that the MS patients had freely recalled fewer objects and resorted to cued recall more often than controls. Language alterations are not common in MS,^5^ thus justifying the absence of differences between groups on the naming task. Naming difficulties are more common in progressive MS^11^ and the present sample contained predominantly patients with relapsing-remittent MS.

Cognitive screening in MS is conditioned by the fact that only a proportion of patients have cognitive deficits, and these can be very diverse. The use of the Phototest can overcome these problems in the cognitive screening of MS, since the instrument evaluates two of the most commonly affected areas: verbal fluency and episodic memory.^22^

The Phototest revealed higher levels of sensitivity and specificity than the MoCA, maybe due to the inclusion of several tasks that showed no significant differences between groups.

The Phototest had a higher value of sensitivity than most of the cognitive screening tests recommended^23^ for MS, namely: the Symbol Digits Modalities Test (91%),^24^ the PASAT (74%),^25^ the Clock Drawing Test (92%)^26^ and the Multiple Sclerosis Neuropsychological Screening Questionnaire (83%).^27^

Phototest specificity is good since it is higher than several screening tests for MS. Good examples are the PASAT,^25^ and the Symbol Digits Modalities Test,^24^ with specificities of 65% and 61%, respectively. However, the specificity proved lower than that of the Clock Drawing Test (specificity 89%).^26^

Performance on the Phototest was not correlated with disease duration, course of disease, medication, disability or fatigue. This finding is in line with previous observations regarding other instruments.^28^ In fact, most of the studies are conflicting regarding the relationship between physical disability and cognitive impairment. While some^29^ confirm the inexistence of a relationship, others^28^ found a weak correlation between neurocognitive functioning and duration of the disease. Moreover, our sample comprised MS patients with low disability status which may have influenced the establishment of this relationship.

None of the screening tests revealed correlation with fatigue. This observation reinforces previous studies pointing to different neuroanatomical bases for fatigue and neurocognition.^30^

Compared to several other screening tests, the Phototest has the advantage of assessing a broader range of cognitive functions in a shorter period of time and does not include paper and pencil tasks. However, the specificity of the Phototest may be limited by the non-inclusion of a subtest that directly assesses speed of information processing, one of the three cognitive domains most commonly affected in MS.^22^

The Phototest is a brief test that is not dependent on motor functions with promising psychometric properties regarding validity, specificity and sensitivity. Clearly the Phototest cannot replace a neuropsychological assessment battery, but may assist in deciding the importance of conducting a more comprehensive assessment of cognitive changes; and may be a key indicator in cases where not much information is required and economic resources and time are scarce.

In direct comparison to the MoCA, the Phototest has major advantages: it is more specific and sensitive to MS; is easier and faster to administer and score; does not require pencil and paper tasks; and is suitable for illiterate patients. 

However, the lack of correlations with MS variables (e.g. disease extent; neurological disability) should be taken into consideration. Future studies should include a higher number of participants with several forms of MS. The relationship between performance on the Phototest, lesion volume and longitudinal fluctuation of the disease should also be explored. 

The present study has several limitations including the small size of the sample, disparity of the groups, the exclusion of illiterate subjects, a constricting inclusion criteria for the control group (participants within 1 SD on MoCA) and the absence of an extensive neuropsychological battery for further concurrent validity. Another limitation of this study was the non-assessment of depressive symptoms, given its impact on cognitive performance.^12^
